# Reversal strategies for vitamin K antagonists in acute intracerebral hemorrhage

**DOI:** 10.1002/ana.24416

**Published:** 2015-05-14

**Authors:** Adrian R. Parry‐Jones, Mario Di Napoli, Joshua N. Goldstein, Floris H. B. M. Schreuder, Sami Tetri, Turgut Tatlisumak, Bernard Yan, Koen M. van Nieuwenhuizen, Nelly Dequatre‐Ponchelle, Matthew Lee‐Archer, Solveig Horstmann, Duncan Wilson, Fulvio Pomero, Luca Masotti, Christine Lerpiniere, Daniel Agustin Godoy, Abigail S. Cohen, Rik Houben, Rustam Al‐Shahi Salman, Paolo Pennati, Luigi Fenoglio, David Werring, Roland Veltkamp, Edith Wood, Helen M. Dewey, Charlotte Cordonnier, Catharina J. M. Klijn, Fabrizio Meligeni, Stephen M. Davis, Juha Huhtakangas, Julie Staals, Jonathan Rosand, Atte Meretoja

**Affiliations:** ^1^ University of Manchester, Manchester Academic Health Sciences Centre, Salford Royal National Health Service Foundation TrustSalfordUnited Kingdom; ^2^ Greater Manchester Neurosciences Centre, Salford Royal National Health Service Foundation TrustSalfordUnited Kingdom; ^3^ Neurological Service, San Camillo de' Lellis General HospitalRietiItaly; ^4^ Neurological Section, Center for Cardiovascular Medicine and Cerebrovascular Disease Prevention (SMDN)SulmonaItaly; ^5^ Department of Emergency MedicineMassachusetts General HospitalBostonMA; ^6^ Department of NeurologyMaastricht University Medical CenterMaastrichtthe Netherlands; ^7^ Department of NeurologyOulu University HospitalOuluFinland; ^8^ Department of NeurologyHelsinki University Central HospitalHelsinkiFinland; ^9^ Department of NeurologyRoyal Melbourne HospitalParkvilleAustralia; ^10^ Department of Neurology and NeurosurgeryRudolf Magnus Brain Center, University Medical Center UtrechtUtrechtthe Netherlands; ^11^ Department of NeurologyUniversity of Lille Nord de France (UDSL), Lille University Hospital CenterLilleFrance; ^12^ Department of NeurologyAustin HospitalHeidelbergAustralia; ^13^ Department of NeurologyUniversity of HeidelbergHeidelbergGermany; ^14^ University College LondonLondonUnited Kingdom; ^15^ Department of Internal MedicineSanta Croce e Carle HospitalCuneoItaly; ^16^ Internal Medicine, Cecina HospitalCecinaItaly; ^17^ Centre for Clinical Brain Sciences, University of EdinburghEdinburghUnited Kingdom; ^18^ Neurointensive Care Unit, Pasteur SanatoriumCatamarcaArgentina; ^19^ Intensive Care Unit, San Juan Bautista HospitalCatamarcaArgentina; ^20^ Department of NeurologyMassachusetts General HospitalBostonMA; ^21^ Department of MedicineImperial College LondonLondonUnited Kingdom; ^22^ Department of Medical EmergencySan Camillo de' Lellis General HospitalRietiItaly; ^23^ Department of Medicine and Florey InstituteUniversity of MelbourneMelbourneAustralia

## Abstract

**Objective:**

There is little evidence to guide treatment strategies for intracerebral hemorrhage on vitamin K antagonists (VKA‐ICH). Treatments utilized in clinical practice include fresh frozen plasma (FFP) and prothrombin complex concentrate (PCC). Our aim was to compare case fatality with different reversal strategies.

**Methods:**

We pooled individual ICH patient data from 16 stroke registries in 9 countries (n = 10 282), of whom 1,797 (17%) were on VKA. After excluding 250 patients with international normalized ratio < 1.3 and/or missing data required for analysis, we compared all‐cause 30‐day case fatality using Cox regression.

**Results:**

We included 1,547 patients treated with FFP (n = 377, 24%), PCC (n = 585, 38%), both (n = 131, 9%), or neither (n = 454, 29%). The crude case fatality and adjusted hazard ratio (HR) were highest with no reversal (61.7%, HR = 2.540, 95% confidence interval [CI] = 1.784–3.616, *p* < 0.001), followed by FFP alone (45.6%, HR = 1.344, 95% CI = 0.934–1.934, *p* = 0.112), then PCC alone (37.3%, HR = 1.445, 95% CI = 1.014–2.058, *p* = 0.041), compared to reversal with both FFP and PCC (27.8%, reference). Outcomes with PCC versus FFP were similar (HR = 1.075, 95% CI = 0.874–1.323, *p* = 0.492); 4‐factor PCC (n = 441) was associated with higher case fatality compared to 3‐factor PCC (n = 144, HR = 1.441, 95% CI = 1.041–1.995, *p* = 0.027).

**Interpretation:**

The combination of FFP and PCC might be associated with the lowest case fatality in reversal of VKA‐ICH, and FFP may be equivalent to PCC. Randomized controlled trials with functional outcomes are needed to establish the most effective treatment. Ann Neurol 2015;78:54–62

Around 20% of all intracerebral hemorrhage (ICH) patients are on vitamin K antagonists (VKA), with the incidence of VKA‐ICH increasing as the population grows older.^1^ The 3‐month case fatality of the condition is high at 50%.^2^, ^3^, ^4^ One‐third of ICH patients develop significant early hematoma expansion,^5^ and this risk is doubled in VKA‐ICH.^6^ Vitamin K takes several hours to initiate sufficient endogenous clotting factor production, so urgent treatments to rapidly replace vitamin K–dependent clotting factors (II, VII, IX, X) are widely used, with the aim of limiting further bleeding. Prothrombin complex concentrate (PCC), fresh frozen plasma (FFP), recombinant factor VIIa, or combinations of these are used, with practice varying between different centers and countries.^7^ Although there is a clear rationale for the use of these agents, none has been conclusively shown to improve outcome after VKA‐ICH.

Evidence from patients with major VKA‐associated bleeding (predominantly gastrointestinal hemorrhage) demonstrates that relative to FFP, PCC normalizes the international normalized ratio (INR) more quickly, reduces the need for red blood cell transfusion, and does not lead to an increase in adverse events.^8^, ^9^ Although PCC is more expensive, it has practical advantages including more rapid administration, smaller infusion volume, and no need for ABO blood type match. This has led to PCC being recommended as a reasonable alternative to FFP in the USA^10^ and the first‐line treatment in the United Kingdom.^11^ The 2014 European consensus‐based ICH guidelines do not provide a recommendation, citing lack of evidence.^12^ Furthermore, different preparations of PCC have different concentrations of the vitamin K–dependent clotting factors, classified as 3‐factor or 4‐factor depending on the concentration of factor VII (FVII). Three‐factor PCCs are widely used in some countries, but may be less effective in correcting the INR than 4‐factor PCC.^13^

Although national and international guidelines recommend clotting factor replacement agents for the treatment of VKA‐ICH, there is currently no definite evidence of benefit and no international consensus. Our aim was to utilize the existing international variation in practice to test for an association between the choice of VKA reversal strategy and survival, adjusted for key prognostic factors, in a large population of patients with VKA‐ICH pooled from 16 registries in Europe, North and South America, and Australia.

## Patients and Methods

### Patients

We performed a retrospective pooled analysis of 16 stroke registries from Argentina, Australia, Finland, France, Germany, Italy, the Netherlands, the United Kingdom, and the USA. Patient registration methods and registration periods varied. Three registries were population‐based, 1 from an international observational study, and 12 from single centers representing both large tertiary teaching hospitals and smaller regional hospitals. Registration of cases was prospective in 11 registries and retrospective in 5. Patient consent was required in 3 registries, there was opt‐out in 3 registries, and the remaining 10 registries were approved as quality registries with consecutive registration of all cases. Patient registration years ranged from 1993 to 2014, with 90% of patients from 2004 to 2013. Registry methods are summarized in Supplementary Table I, with further details previously published.^2^, ^3^, ^4^, ^14^, ^15^, ^16^, ^17^, ^18^, ^19^, ^20^

According to a prespecified protocol, we included patients aged ≥18 years taking any VKA at the time of their ICH. We excluded patients with ICH secondary to trauma or tumor, primary subarachnoid hemorrhage, or hemorrhagic transformation of ischemic stroke, and those with baseline INR < 1.3. We also excluded those with missing data on reversal therapy received or variables used for adjustment: age, gender, INR, Glasgow Coma Scale (GCS), and imaging parameters (infratentorial location, intraventricular extension, baseline ICH volume). We prespecified exclusion of patients treated >24 hours from ICH onset, but later added these cases following a request from a manuscript reviewer.

### Procedures

The registries provided data for the pooled analysis using a standardized form. Baseline volume was defined as the volume of intraparenchymal hemorrhage on the first scan, excluding any intraventricular hemorrhage (IVH). ICH volume was estimated with the ABC/2 method^21^ in 12 and with planimetric methods in 4 registries. IVH volume was estimated with planimetric methods (2 registries), Hallevi score^22^ (3 registries), or an estimate of the Hallevi score based on the Graeb score multiplied by 2 (6 registries). Immediate palliation was defined as the decision to withhold active treatment and to provide palliative care only immediately after ICH diagnosis. Therefore, patients who received either FFP or PCC were, by definition, not immediately palliated. Patients were classified into treatment arms of "FFP alone" (FFP but no PCC), "PCC alone" (PCC but no FFP), "combination" (both FFP and PCC), and "no reversal" (neither FFP nor PCC). PCC preparations were further classified as 4‐factor PCC or 3‐factor PCC according to the presence or absence of FVII. Patients receiving both 3‐factor PCC and FVIIa were considered to have received 4‐factor PCC. We also recorded whether patients were given vitamin K. Our primary outcome measure was all‐cause case fatality by 30 days or end of follow‐up if earlier.

### Statistical Analysis

Statistical analyses were performed according to a prespecified protocol. For all descriptive analyses, we compared patient characteristics by treatment arm using the Kruskal–Wallis and chi‐square tests, as appropriate. For the outcome of case fatality by 30 days, Kaplan–Meier estimates and the log‐rank test were used for univariate analysis and Cox proportional hazard models for multivariate analysis, with the multivariate comparison predefined as the primary outcome measure. We adjusted for prespecified baseline characteristics known to be associated with case fatality in ICH: age, log‐transformed ICH volume, INR, and GCS as continuous variables, and sex, infratentorial location, and intraventricular extension as binary variables.^10^ We confirmed the assumption of proportional hazards by visual examination of the log (minus log) curves. Hazard ratios (HRs) with 95% confidence intervals (CIs) are reported, together with a survival plot at covariate means.

To examine for potential heterogeneity in the association between treatment and all‐cause case fatality, we estimated this in several subgroups with prespecified cutoffs using the same Cox model, including a treatment by subgroup interaction term. As sensitivity analysis, we performed a prespecified propensity score–matched analysis. The propensity score for receiving the combination treatment was estimated using the same variables as in the primary analysis. We then matched patients in all 4 treatments arms using the nearest neighbor method with caliper set at 0.2 standard deviations of the logit of the propensity score. The primary analysis was duplicated in the propensity score–matched population.

Our study was powered for the primary outcome only. Assuming 50% case fatality by 30 days in the no reversal arm, and 25% HR reduction with any of the active treatments, we would have 80% power to detect this difference with 1,087 patients. The 2‐sided threshold for statistical significance was set at *p* = 0.05, with no correction for multiple comparisons justified by the hypothesis‐generating exploratory nature of this analysis.

## Results

**Figure 1:**
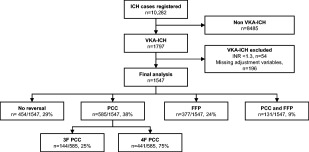
Study flowchart depicting exclusions and treatment strategies used in patients included in the final analysis. F = factor; FFP = fresh frozen plasma; ICH = intracerebral hemorrhage; INR = international normalized ratio; PCC = prothrombin complex concentrate; VKA = vitamin K antagonists.

The pooled registries contained 10,282 ICH patients over the study period, and after exclusions 1,547 were included in the analysis (Fig 1, Supplementary Table II). The reversal strategies were FFP alone (n = 377, 24%), PCC alone (n = 585, 38%), combination (n = 131, 9%), or no reversal (n = 454, 29%; Table 1). Whereas PCC was the dominant treatment elsewhere, 3 centers preferred FFP (Boston, Catamarca, and Cuneo), and the Australian centers combined both. Of patients treated with PCC alone, 144 (25%) received 3‐factor PCC and 441 (75%) received 4‐factor PCC. Of the patients receiving the combination therapy, 127 (97%) received 3‐factor PCC. Baseline characteristics were significantly different between the treatment groups, except for age and infratentorial location (see Table 1). No patients died before planned reversal therapy was given.

**Table 1:** Baseline Characteristics by Treatment Group

Characteristic	Total Cohort, n = 1,547	No Reversal, n = 454	FFP Only, n = 377	PCC Only, n = 585	FFP + PCC, n = 131	*p*
Age, yr	77 (70–83)	78 (71–84)	78 (71–83)	77 (70–82)	76 (68–84)	0.159
Male sex	884 [57]	239 [53]	217 [58]	342 [58]	86 [66]	0.045
GCS at admission	13 (8–15)	12 (5–15)	13 (6–15)	14 (11–15)	14 (13–15)	<0.001
Baseline blood glucose, mg/dl^a^	141 (115–175)	152 (119–191)	139 (112–172)	137 (114–166)	141 (116–178)	0.001
Baseline ICH volume, ml	18 (6–52)	28 (7–79)	16 (5–52)	15 (5–40)	18 (6–34)	<0.001
Infratentorial location	272 [18]	77 [17]	59 [16]	106 [18]	30 [23]	0.288
Intraventricular extension	757 [49]	241 [53]	203 [54]	263 [45]	50 [38]	<0.001
Baseline IVH volume, ml^a^	0 (0–8)	0 (0–25)	0 (0–11)	0 (0–5)	0 (0–3)	<0.001
INR at admission	2.9 (2.4–3.7)	2.8 (2.2–3.7)	2.9 (2.3–3.5)	3.0 (2.5–3.8)	2.9 (2.4–3.5)	0.024
Onset‐to‐treatment, min^a^	305 (175–660)	—	340 (203–630)	285 (165–625)	355 (185–845)	0.077
Acute intracranial surgery	144/1,536 [9]	14/449 [3]	37/377 [10]	73/580 [13]	20/130 [15]	<0.001
Received vitamin K	1,024/1,477 [69]	137/451 [30]	330/366 [90]	434/530 [82]	123/130 [95]	<0.001

All values are median (interquartile range) or No. [%].

a Data for glucose/IVH volume/onset‐to‐treatment time missing in 156/217/0 of no reversal, 43/47/305 of FFP, 124/111/188 of PCC, and 46/7/6 of FFP + PCC patients, respectively.

FFP = fresh frozen plasma; GCS = Glasgow Coma Scale; ICH = intracerebral hemorrhage; INR = international normalized ratio; IVH = intraventricular hemorrhage; PCC = prothrombin complex concentrate.

**Figure 2:**
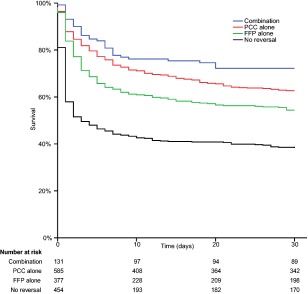
Kaplan–Meier survival analysis of 30‐day survival after intracerebral hemorrhage stratified by treatment strategy. FFP = fresh frozen plasma; PCC = prothrombin complex concentrate. [Color figure can be viewed in the online issue, which is available at www.annalsofneurology.org.]

All‐cause case fatality by 30 days was 45.7% (95% CI = 43.2–48.2%) overall and varied markedly by treatment arm, being 61.7% (95% CI = 57.2–66.2%) with no reversal, 45.6% (95% CI = 40.5–50.7%) with FFP, 37.3% (95% CI = 33.3–41.2%) with PCC, and 27.8% (95% CI = 20.1–35.5%) with the combination therapy (Fig 2). Information on immediate palliation was missing in a large proportion of the patients without reversal, but in the 277 with these data recorded, 142 (51%) were immediately palliated.

**Figure 3:**
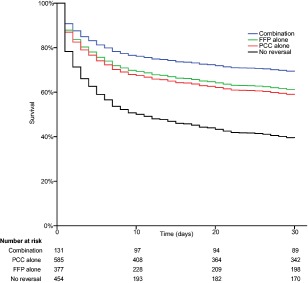
Cox regression survival curves for 30‐day survival after intracerebral hemorrhage (ICH) stratified by treatment strategy, adjusting for age, sex, ICH volume, infratentorial location, intraventricular extension, baseline international normalized ratio, and Glasgow Coma Scale. FFP = fresh frozen plasma; PCC = prothrombin complex concentrate. [Color figure can be viewed in the online issue, which is available at www.annalsofneurology.org.]

**Figure 4:**
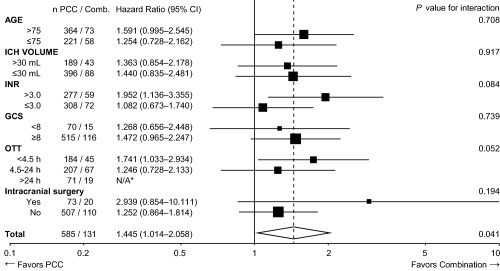
Forest plot showing adjusted hazard ratios for case fatality rate by 30 days according to age, hematoma volume, baseline international normalized ratio (INR), baseline Glasgow Coma Scale (GCS), intracranial surgery, and onset‐to‐treatment time (OTT). *Cox regression coefficients fail to converge with 0/19 deaths in the combination group, 18/71 in the prothrombin complex concentrate (PCC) group. CI = confidence interval; Comb. = combination therapy; ICH = intracerebral hemorrhage; N/A = not applicable.

After adjusting for baseline imbalances using Cox regression, the HR for death within 30 days of ICH was the highest with no reversal (HR = 2.540, 95% CI = 1.784–3.616, *p* < 0.001), followed by PCC alone (HR = 1.445, 95% CI = 1.014–2.058, *p* = 0.041), and FFP alone (HR = 1.344, 95% CI = 0.934–1.934, *p* = 0.112), when compared to combination therapy (Table 2, Fig 3). Outcomes with PCC versus FFP were similar (HR = 1.075, 95% CI = 0.874–1.323, *p* = 0.492). Within the PCC group, 4‐factor PCC use was associated with higher case fatality compared to 3‐factor PCC (HR = 1.441, 95% CI = 1.041–1.995, *p* = 0.027; Table 3). The treatment effect did not vary in any of the prespecified subgroups (Fig 4), but was borderline nonsignificant after post hoc limiting to patients not initially palliated (HR = 1.586, 95% CI = 0.994–2.531, *p* = 0.053, for combination vs no reversal).

**Table 2:** Cox Regression Model for 30‐Day Case Fatality after Intracerebral Hemorrhage (n = 1,547)

Factor	Univariate HR (95% CI)	*p*	Multivariate HR (95% CI)	*p*
Age, per year	1.016 (1.008–1.024)	<0.001	1.020 (1.012–1.029)	<0.001
Male sex	1.029 (0.886–1.196)	0.707	1.172 (1.005–1.366)	0.043
ICH volume, per log transformed ml	1.889 (1.766–2.020)	<0.001	1.429 (1.327–1.539)	<0.001
Infratentorial location	1.020 (0.839–1.241)	0.842	1.386 (1.125–1.707)	0.002
Intraventricular extension	3.139 (2.672–3.687)	<0.001	1.682 (1.410–2.007)	<0.001
Baseline INR, per unit	1.108 (1.071–1.147)	<0.001	1.052 (1.012–1.094)	0.010
Glasgow Coma Scale, per point	0.822 (0.808–0.836)	<0.001	0.878 (0.860–0.896)	<0.001
Combination of FFP and PCC	Reference		Reference	
PCC alone	1.407 (0.989–2.002)	0.058	1.445 (1.014–2.058)	0.041
FFP alone	1.849 (1.291–2.649)	<0.001	1.344 (0.934–1.934)	0.112
No reversal	3.182 (2.248–4.505)	<0.001	2.540 (1.784–3.616)	<0.001

CI = confidence interval; FFP = fresh frozen plasma; HR = hazard ratio; ICH = intracerebral hemorrhage; INR = international normalized ratio; PCC = prothrombin complex concentrate.

**Table 3:** Cox Regression Model for 30‐Day Case Fatality after Intracerebral Hemorrhage in Patients Treated with PCC Alone (n = 585)

Factor	Univariate HR (95% CI)	*p*	Multivariate HR (95% CI)	*p*
Age, per year	1.013 (0.999–1.027)	0.077	1.022 (1.006–1.037)	0.005
Male sex	1.040 (0.792–1.366)	0.777	1.202 (0.910–1.587)	0.196
ICH volume, per log transformed ml	1.725 (1.528–1.946)	<0.001	1.538 (1.345–1.758)	<0.001
Infratentorial location	1.169 (0.834–1.637)	0.365	1.534 (1.068–2.202)	0.020
Intraventricular extension	3.510 (2.630–4.684)	<0.001	2.338 (1.720–3.178)	<0.001
Baseline INR, per unit	1.089 (1.003–1.182)	0.043	1.064 (0.978–1.157)	0.147
Glasgow Coma Scale, per point	0.843 (0.815–0.872)	<0.001	0.905 (0.869–0.943)	<0.001
3‐factor PCC, n = 144	Reference		Reference	
4‐factor PCC, n = 441	1.147 (0.832–1.581)	0.403	1.441 (1.041–1.995)	0.027

CI = confidence interval; HR = hazard ratio; ICH = intracerebral hemorrhage; INR = international normalized ratio; PCC = prothrombin complex concentrate.

As surgery rates and vitamin K use varied between the treatment groups, and baseline glucose and IVH volume have been suggested to be associated with outcome, we ran a post hoc analysis of the primary outcome, now adding these into the adjusted model (n = 978). The HR changed little, being 2.424 (95% CI = 1.505–3.905, *p* < 0.001) for no reversal, 1.624 (95% CI = 1.051–2.510, *p* = 0.029) for PCC, and 1.298 (95% CI = 0.837–2.014, *p* = 0.244) for FFP compared to the combination therapy. As treatments were clustered by center, we also ran a second post hoc analysis, now introducing a random effect for center (shared frailty) into the primary analysis. Again the HR changed little, being 3.370 (95% CI = 2.195–5.173, *p* < 0.001) for no reversal, 1.822 (95% CI = 1.122–2.961, *p* = 0.015) for FFP, and 1.682 (95% CI = 1.090–2.594, *p* = 0.019) for PCC compared to combination therapy.

Finally, we performed sensitivity analyses of the primary outcome in a propensity score–matched population of 131 patients from each treatment arm well balanced on baseline characteristics (Supplementary Table III). In this analysis, there was also higher case fatality with no reversal (HR = 2.686, 95% CI = 1.764–4.088, *p* < 0.001), PCC alone (HR = 1.544, 95% CI = 1.000–2.386, *p* = 0.050), and FFP alone (HR = 1.149, 95% CI = 0.727–1.816, *p* = 0.551), compared to the combination therapy (Supplementary Table IV).

## Discussion

Our international, multicenter, observational study of 1,547 VKA‐ICH patients demonstrates considerable variation in the practice of anticoagulation reversal after acute ICH. Most centers used PCC alone and some centers used solely FFP, whereas Australian centers systematically combined both. Treatment with any clotting factor product was associated with less than half the adjusted risk of death by 30 days. When comparing different clotting factor products, we found no significant difference between PCC and FFP. Case fatality was lower in patients treated with a combination of PCC and FFP, when compared to either alone. For those receiving PCC alone, we found that administration of 3‐factor PCC was associated with improved survival compared to treatment with 4‐factor PCC.

An ongoing phase 2 trial (INCH; NCT00928915) is testing FFP versus PCC in 74 patients with correction of INR as the primary outcome,^23^ but has not yet reported. A recent German observational study on VKA‐OAC (n = 1,176), published after the submission of our study, suggested that rapid reversal of INR is associated with less ICH expansion and lower in‐hospital mortality, but was not powered to compare reversal therapies due to a uniform national practice of using PCC.^24^ Previous observational studies comparing treatments have been small (17–181 patients), with variable results with regard to case fatality,^14^, ^25^, ^26^ functional outcome,^27^, ^28^ and hematoma expansion.^29^ These studies also combined patients receiving FFP with either PCC^26^, ^29^ or no reversal,^14^, ^23^, ^27^ precluding direct comparison of specific reversal strategies.

Our data show that receiving no clotting factor replacement is associated with more than twice the risk of death by 30 days, when compared to all 3 treated groups. This might be expected, as patients without reversal are a select group, usually palliated early due to a perceived dismal prognosis. However, it is possible that reversal treatment does reduce mortality, given that we found similar results in our propensity score analysis. Our study had sufficient power to compare survival in those receiving FFP and PCC. The finding that patients with either therapy have an equivalent adjusted risk of death in our large cohort is thus an important and novel finding.

The observed trend toward a reduced adjusted risk of death in patients receiving a combination of FFP and PCC must be interpreted in the context of our observational study design. This group was relatively small (131 patients vs >350 patients in each of the other 3 groups), and it is of note that the combination group had slightly higher rates of surgery (15% vs 10–13%). Surgery was not adjusted for in our prespecified Cox regression model, given the existing uncertainty around the impact of surgery on outcome.^10^, ^12^, ^30^ However, adding surgery into the model post hoc did not markedly change our findings. The combination group consisted largely of patients from 2 Australian centers; thus, there may be unmeasured confounding factors related to the characteristics of the local population or aspects of clinical care that could account for the observed difference. It is also possible that factors present in FFP such as FXIII, fibrinogen, antithrombin, and von Willebrand factor may confer additional benefit when combined with PCC. For example, FXIII is present in FFP (but not PCC) and creates fibrin cross links, making clots more stable. A drop in FXIII activity has previously been associated with reduced hematoma growth in patients not on VKA, suggesting that consumption of FXIII stabilizes clots.^31^ FFP also contains endogenous inhibitors of fibrinolysis (eg, plasminogen activator inhibitor 1 and 2). Our finding that combination treatment was associated with a lower risk of death remained robust in all the prespecified subgroups (see Fig 4).

It is more difficult to interpret the finding that among patients receiving PCC only, 3‐factor PCC was associated with lower case fatality compared to 4‐factor PCC. That patients who received the combination therapy also had 3‐factor PCC as their PCC product raises the possibility that there is a true difference between different PCC products. In patients taking warfarin, suppression of prothrombin (FII) activity levels seems to be more important for sustained antithrombotic efficacy than suppression of other vitamin K–dependent clotting factors.^32^ Thus, administration of 4‐factor PCC with significant FVII content may not be essential to reducing further bleeding and could increase thrombotic complications. The compositions of the multiple PCC products on the market also vary by heparin, antithrombin, and protein C and S concentrations, which all may be relevant. We did not have sufficient numbers to compare individual products, or collect data on thrombotic complications or causes of death, so further studies are required to test this hypothesis.

Our study benefits from a large sample size of 1,547 patients. The patients included in the study are representative of routine clinical care in varied health care systems across a range of countries. The majority of patients presented between 2004 and 2013 and thus are more closely representative of current practice than cohorts described in previous studies. All patients included in our analysis had key prognostic factors available describing age, level of consciousness on presentation, imaging findings, and baseline INR, allowing us to adjust for these in our multivariate models.

There are also some potential weaknesses inherent in our observational study design. Our results might be limited by bias related to unmeasured or hidden covariates. Allocation to treatment group was dictated by the preferences of the attending physician in consultation with the patient and/or their family, guided by local and national policies. One of the main determinants of outcome in ICH is the decision to palliate early, which often forms a so‐called self‐fulfilling prophecy.^10^ Half of the patients without any reversal fell into this category. The decision not to treat patients may also be influenced by unmeasured factors, such as poor premorbid health or frailty, and these may contribute to the observed association between poor outcome and no treatment. It is possible that other factors specific to each center (such as the use of other interventions and level of supportive care, or ethnic composition) could also have influenced survival. As each center followed a certain treatment strategy and thus the center and treatment effects were strongly correlated, we performed a post hoc shared frailty analysis, which within its limitations suggested that the differences in outcome were not explained by center‐specific differences. Although our data set is large, the numbers in each treatment arm are still relatively small, and we thus risk both overmodeling and type II errors. As our observational study can only be considered hypothesis generating, this is not a major risk. The methodology of ICH volume estimation differed across the centers but is unlikely to be a major source of error, as the ABC/2 and planimetric methods used produce similar results in regular and irregular bleeds alike.^21^ Estimates of IVH volume varied and were missing in many patients, as were data on baseline blood glucose. Also, we did not adjust for other factors that have been variably associated with outcome in ICH, such as blood pressure or treatment delays, as they were not in our statistical analysis plan and not available. Finally, we do not have data describing functional outcomes, and thus do not know whether the association of improved survival with treatment is at the expense of a greater proportion of patients living with severe disability.

Our findings support the hypothesis that the use of clotting factor replacement therapy in VKA‐ICH is associated with improved survival. Although some guidelines recommend PCC over FFP based on limited evidence and expert opinion,^11^ we found no evidence to support this policy. The clinical significance of our preliminary finding of improved survival in those treated with combination therapy, or with 3‐factor PCC compared to 4‐factor PCC, requires further investigation. Based on our findings, we would recommend prompt clotting factor replacement for VKA‐ICH, with the choice of preparation guided by local policy. A definitive randomized controlled trial testing the effect of PCC, FFP, and combination therapy on functional outcomes is urgently needed to resolve the existing uncertainty regarding the best reversal strategy for VKA‐ICH. It has been previously estimated that a population base of around 67 million would be required to conduct a 5‐year trial of this nature,^33^ so such a trial would be a major undertaking, although the effect sizes noted in our observational study and adaptive or 3‐arm trial designs may make such a trial more feasible.

## Authorship

A.R.P.‐J., M.D.N., and A.M. conceived the study, pooled and analyzed the data, and drafted the manuscript. All authors contributed to the design of the study, collection and interpretation of the data, and editing of the manuscript.

## Potential Conflicts of Interest

J.N.G.: grant, consultancy, CSL Behring. F.H.B.M.S.: grants, Center for Translational Molecular Medicine, Dutch Heart Foundation. T.T.: advisory board, Boehringer Ingelheim (payments to institution), Mitsubishi Pharma (payments to institution), Bayer, Pfizer; consultancy/national coordinator and PI (payments to institution), Lundbeck, Sanofi Aventis; National coordinator and PI (payments to institution), PhotoThera, BrainsGate; steering committee chairman, national coordinator, and PI (payments to institution), Mitsubishi Pharma; consultancy (payments to institution), Orion Pharma; PI (payments to institution), Bayer, Pfizer; speaking fees, Professio Finland. R.V.: speaking fees, consultancy, Bayer, Boehringer, BMS, Daiichi Sankyo, Roche; research support, Bayer, Boehringer. C.C.: advisory board, Bayer (honoraria paid to Adrinord); travel expenses, Teva; investigator honoraria, Pfizer (paid to Lille University and Adrinord). S.M.D.: advisory board, Boehringer Ingelheim; travel expenses, BMS Pfizer, Allergan, Covidien, EVER Neuropharma. J.H.: speaking fee, Sanquin Oy. J.R.: consultancy, Boehringer Ingelheim. A.M.: honoraria, travel expenses, Siemens.

## Supporting information

Supporting Information.

Supporting Information Figure 1.

Supporting Information Figure 2.
