# Supplementary material for: Reversal strategies for vitamin K antagonists in acute intracerebral hemorrhage
Source: Ann Neurol. 2015 May 14;78(1):54–62. doi: 10.1002/ana.24416 (PMC4654243; doi:10.1002/ana.24416)
Supplement: Supplementary file 1 — Supporting Information. [file ana0078-0054-sd1.pdf]

SUPPLEMENTAL MATERIAL

Supplement to: Parry-Jones AR, Di Napoli M, Goldstein JN, Schreuder FHBM, Tetri S, Tatlisumak T, Yan B, van Nieuwenhuizen K, Dequatre-Ponchelle N, Lee-Archer M, Horstmann S, Wilson D, Pomero F, Masotti L, Lerpiniere C, Godoy DA, Cohen AS, Houben R, Al-Shahi Salman R, Pennati P, Fenoglio L, Werring D, Veltkamp R, Wood E, Dewey HM, Cordonnier C, Klijn CJM, Meligeni F, Davis SM, Huhtakangas J, Staals J, Rosand J, Meretoja A. Reversal strategies for vitamin K antagonists in acute intracerebral hemorrhage.

Contents

Table I. Registries included in the study.

Table II. Baseline characteristics, treatment, and outcome of included and excluded patients

Table III. Baseline characteristics of the propensity score matched population.

Table IV. Cox regression model for 30-day case-fatality after intracerebral hemorrhage in the propensity score matched cohort (n=524).

Table I. Registries included in the study.

Hospital or Registry	Location	Hospital or Population Based	Study design	Patient consent for registration or not required as approved as a quality register	Period of patient series	Date of patient series end for this data extraction	Local specific exclusions to registration	Vitamin K antagonist-related ICH among all ICH patients registered during study period, n	Patients included in present analysis, n, broken down by treatment groups†
Sanatorio Pasteur	Catamarca, Argentina	1 Hospital	Prospective	Not required	1.2.2006	1.1.2014	Patients admitted >24 h from onset	17/187 (9%)	17 (100%)<br>0 / 12 / 2 / 3
Austin Hospital	Melbourne, Australia	1 Hospital	Prospective	Not required	1.1.2006	19.9.2013	None	69/480 (14%)	60 (87%)<br>7 / 0 / 13 / 40
The Royal Melbourne Hospital*	Melbourne, Australia	1 Hospital	Prospective	Not required	1.10.2007	31.12.2013	None	119/668 (18%)	117 (98%)<br>23 / 2 / 13 / 79
Helsinki University Central Hospital	Province of Helsinki and Uusimaa, Finland	1 Hospital	Retrospective	Not required	1.1.2005	31.3.2010	None	132/1013 (13%)	130 (98%)<br>51 / 26 / 49 / 4
Oulu ICH Study*	Northern Ostrobothnia, Finland	Population	Retrospective	Not required	1.1.1993	31.12.2008	Vascular malformations, hematologic malignancy, hemophilia	182/982 (19%)	163 (90%)<br>112 / 11 / 40 / 0
Centre Hospitalier Régional Universitaire de Lille	Lille, France	1 Hospital	Prospective	Not required	1.11.2004	30.4.2009	Vascular malformations	87/562 (15%)	75 (86%)<br>52 / 0 / 23 / 0
University of Heidelberg	Heidelberg city and surroundings, Germany	1 Hospital	Prospective	Yes	31.8.2009	28.2.2011	1 patient did not consent to study participation	51/206 (25%)	45 (88%)<br>4 / 6 / 35 / 0
San Camillo de'Lellis General Hospital	Province of Rieti, Italy	Population	Prospective	Not required	1.1.2008	31.12.2013	None	88/637 (14%)	84 (95%)<br>35 / 8 / 38 / 3
Cecina Hospital	Cecina, Italy	1 Hospital	Retrospective	Not required	1.1.2006	31.8.2013	None	32/170 (19%)	32 (100%)<br>3 / 1 / 26 / 2
S. Croce e Carle' Hospital	Cuneo, Italy	1 Hospital	Retrospective	Yes	1.1.2005	31.12.2010	None	34/274 (12%)	34 (100%)<br>8 / 26 / 0 / 0
Maastricht University Medical Center	South Limburg, the Netherlands	3 Hospitals	Retrospective	Opt Out	1.1.2004	31.12.2009	None	290/1252 (23%)	248 (86%)<br>69 / 0 / 179 / 0
University Medical Center Utrecht*	Utrecht, the Netherlands	1 Hospital	Prospective	Opt out	1.1.2007	31.7.2012	Vascular malformations	81/405 (20%)	65 (80%)<br>12 / 0 / 53 / 0
Salford Royal Hospital	Manchester, UK	1 Hospital	Prospective	Not required	1.1.2008	31.1.2014	None	56/633 (9%)	55 (98%)<br>8 / 0 / 47 / 0
Clinical Relevance Of Microbleeds In Stroke (CROMIS 2) Study	UK with one centre in the Netherlands	Multiple Hospitals	Prospective	Yes	1.8.2011	1.10.2013	None	172/735 (23%)	93 (54%)<br>41 / 0 / 52 / 0
Lothian Audit of the Treatment of Cerebral Haemorrhage (LATCH)	Lothian Healthboard, Edinburgh, Scotland. UK	Population	Prospective	Opt Out	1.6.2010	31.5.2012	None	36/350 (10%)	28 (78%)<br>13 / 0 / 15 /0
Massachusetts General Hospital*	Boston, Massachusetts, USA	1 Hospital	Prospective	Not required	1.1.2001	29.4.2014	None	351/1728 (20%)	301 (86%)<br>16 / 285 / 0 / 0
Total								1797/10282 (17%)	1547 (86%)<br>454 / 377 / 585 / 131

*These four registries used planimetric methods for ICH volume estimate. The other 12 registries used the ABC/2 method.

† No reversal / fresh frozen plasma / prothrombin complex concentrate / combination of fresh frozen plasma and prothrombin complex concentrate

Table II. Baseline characteristics, treatment, and outcome of included and excluded patients

	Included n=1547		Excluded n=250		*P*
	Missing data, n	Data	Missing data, n	Data	
Age	0	77 (70-83)	12	76 (68-83)	0.287
Male sex	0	884 (57%)	16	144 (62%)	0.205
GCS at admission	0	13 (8-15)	43	14 (9-15)	<0.001
Baseline blood glucose, mg/dl	369	141 (115-175)	96	135 (110-169)	0.259
Baseline ICH volume, mL	0	18 (6-52)	46	15 (3-41)	0.002
Infratentorial hemorrhage	0	272 (18%)	28	43 (19%)	0.515
Intraventricular extension	0	757 (49%)	39	88 (42%)	0.049
Baseline IVH volume, mL	382	0 (0-8)	73	(0-3)	0.003
INR at admission	0	2.9 (2.4-3.7)	65	2.2 (1.2-3.3)	0.072
Onset-to-treatment, min	498	305 (175-660)	141	483 (298-5443)	<0.001
Received vitamin K	70	1024 (69%)	58	53 (28%)	<0.001
Acute intracranial surgery	11	144 (9%)	32	12 (6%)	0.060
Reversal therapy	0		55		<0.001
No reversal		454 (29%)		105 (54%)	
FFP only		377 (24%)		37 (19%)	
PCC only		585 (38%)		52 (27%)	
Combination of FFP and PCC		131 (9%)		1 (1%)	
Case-fatality during 30-day follow-up	0	701 (45%)	112	68 (49%)	<0.001

All values are median (interquartile range) or n (%).

GCS indicates Glasgow Coma Scale; ICH, intracerebral hemorrhage; INR, international normalized ratio; FFP, fresh frozen plasma; PCC, prothrombin complex concentrate.

Table III. Baseline characteristics of the propensity score matched population.

	Total cohort n=524	No reversal n=131	FFP only n=131	PCC only n=131	FFP + PCC N=131	*P*
Age	77 (69-83)	76 (70-82)	77 (69-82)	77 (70-82)	76 (68-84)	0.883
Male sex	337 (64%)	84 (64%)	90 (69%)	77 (59%)	86 (66%)	0.399
GCS at admission	14 (12-15)	14 (12-15)	15 (13-15)	14 (12-15)	14 (13-15)	0.012
Baseline blood glucose, mg/dl	139 (115-173)	144 (119-171)	133 (108-170)	139 (119-173)	141 (116-178)	0.533
Baseline ICH volume, mL	16 (6-40)	17 (5-49)	13 (6-34)	17 (7-36)	17 (5-34)	0.264
Infratentorial hemorrhage	121 (23%)	30 (23%)	29 (22%)	32 (24%)	30 (23%)	0.977
Intraventricular extension	201 (38%)	51 (39%)	54 (41%)	46 (35%)	50 (38%)	0.787
Baseline IVH volume, mL	0 (0-4)	0 (0-6)	0 (0-4)	0 (0-5)	0 (0-3)	0.740
INR at admission	2.9 (2.3-3.5)	2.7 (2.2-3.6)	2.8 (2.2-3.5)	2.9 (2.4-3.8)	2.9 (2.4-3.5)	0.141
Onset-to-treatment, min*	333 (180-690)	-	315 (194-630)	274 (165-625)	355 (185-845)	0.197
Received vitamin K	372/507 (73%)	35/131 (27%)	113/126 (90%)	101/120 (90%)	123/130 (95%)	<0.001
Acute intracranial surgery	51/521 (10%)	5/131 (4%)	11/131 (8%)	15/129 (12%)	20/130 (15%)	0.014

All values are median (interquartile range) or n (%). Hosmer Lemeshow p=0.955 for the propensity score model. C-statistic: 0.633 (95%CI 0.585-0.682).
*Data available only for 22/131 FFP, 85/131 PCC, and 125/131 FFP + PCC patients.
GCS indicates Glasgow Coma Scale; ICH, intracerebral hemorrhage; INR, international normalized ratio; FFP, fresh frozen plasma; PCC, prothrombin complex concentrate.

Table IV. Cox regression model for 30-day case-fatality in the propensity score matched cohort (n=524).

	Univariate HR (95% CI)	*P*	Multivariable HR (95% CI)	*P*
Age, per year	1.022 (1.006-1.038)	0.005	1.022 (1.005-1.039)	0.010
Male sex	0.913 (0.680-1.226)	0.543	1.268 (0.931-1.726)	0.131
ICH volume, per log transformed mL	2.118 (1.841-2.437)	<0.001	1.559 (1.326-1.833)	<0.001
Infratentorial location	0.799 (0.555-1.149)	0.225	1.163 (0.784-1.726)	0.452
Intraventricular extension	2.993 (2.235-4.009)	<0.001	1.782 (1.289-2.464)	<0.001
Baseline INR, per unit	1.134 (1.040-1.237)	0.005	1.052 (0.965-1.146)	0.251
Glasgow Coma Scale, per point	0.823 (0.796-0.850)	<0.001	0.883 (0.846-0.922)	<0.001
Reversal strategy				
Combination of FFP and PCC	Reference		Reference	
PCC alone	1.387 (0.901-2.137)	0.138	1.544 (1.000-2.386)	0.050
FFP alone	1.105 (0.703-1.739)	0.665	1.149 (0.727-1.816)	0.551
No reversal	2.250 (1.497-3.382)	<0.001	2.686 (1.764-4.088)	<0.001

HR indicates hazard ratio; ICH, intracerebral hemorrhage; FFP, fresh frozen plasma; PCC, prothrombin complex concentrate.
